# Rickettsioses in Brazil: distinct diseases and new paradigms for epidemiological surveillance

**DOI:** 10.1590/0037-8682-0732-2020

**Published:** 2021-02-10

**Authors:** Álvaro A. Faccini-Martínez, Felipe da Silva Krawczak, Stefan Vilges de Oliveira, Marcelo Bahia Labruna, Rodrigo Nogueira Angerami

**Affiliations:** 1 Asociación Colombiana de Infectología, Committee of Tropical Medicine, Zoonoses and Travel Medicine, Bogotá, Colombia.; 2 Universidade Federal de Goiás, Escola de Veterinária e Zootecnia, Setor de Medicina Veterinária Preventiva, Goiânia, GO, Brasil.; 3 Universidade Federal de Uberlândia, Faculdade de Medicina, Departamento de Saúde Coletiva, Uberlândia, MG, Brasil.; 4 Universidade de São Paulo, Faculdade de Medicina Veterinária e Zootecnia, Departamento de Medicina Veterinária Preventiva e Saúde Animal, São Paulo, SP, Brasil.; 5 Universidade Estadual de Campinas, Hospital de Clínicas, Seção de Epidemiologia Hospitalar, Campinas, SP, Brasil.

Dear Editor:

Rickettsioses are a topic of increasing relevance in public health globally^1^. New species, new diseases, and changes in epidemiological scenarios are some of the factors that have imposed the need for new approaches toward rickettsioses in relation to patient care and preventive, control, and surveillance activities.

Brazilian spotted fever (BSF), caused by *Rickettsia rickettsii*, has been a nationally notifiable disease in Brazil since 2001. It should be understood as a paradigm of reemerging disease, notably from the 1980s when it reappeared in the metropolitan region of São Paulo city and Campinas and São João da Boa Vista regions in the inner São Paulo state^2^. Since then, BSF cases have been increasingly reported in a number of Brazilian states, with a particularly high incidence in the southeastern region, in the states of São Paulo and Minas Gerais^2^. However, since the first reports of BSF cases in the Santa Catarina state (southern region) in 2003, a new scenario has been observed, raising new challenges to health services and discussions in the academic field^3^.

While BSF in the southeastern region prevails as a multisystem disease with a high frequency of hemorrhagic manifestations and organ dysfunction and, consequently, high fatality rates (approximately 55%), in the Santa Catarina state (southern region) the disease has been characterized by fever with non-specific systemic clinical manifestations of benign character and no fatalities have been reported to date^2^ ^,^ ^3^. Additionally, the high frequency of two clinical signs present in BSF cases in Santa Catarina, but not in endemic areas of the southeastern region, deserves mention-a characteristic skin lesion at the tick bite site (the inoculation eschar) and ipsilateral lymphadenopathy^3^ ^,^ ^4^. 

Based on the clinical profile of BSF in Santa Catarina, particularly because of the zero fatality rate and low morbidity, an inevitable hypothesis was raised-the causative agent was either a less virulent strain of *R. rickettsii* or another *Rickettsia* species^3^. All “atypical” benign cases in that state had been confirmed by the serological criteria using an indirect immunofluorescence assay without confirmation by microbiological isolation or molecular tools such as polymerase chain reaction^3^. Nevertheless, in 2015, 6 years after the above hypothesis was raised, the *R. parkeri* strain Atlantic rainforest was molecularly identified for the first time as the etiological agent of a spotted fever case in Santa Catarina. The patient presented with mild clinical signs, inoculation eschar, and seroconversion to spotted fever group rickettsiae, including antigens of both *R. rickettsii* and *R. parkeri* ^5^. Several field studies confirmed a new epidemiological scenario for tick-borne spotted fever. Tick species different from the classical vectors of *R. rickettsii* were implicated in sustaining a natural cycle of *R. parkeri* strain Atlantic rainforest involving domestic dogs and wild small rodents^4^.

In Brazil, 2,127 laboratory-confirmed cases of BSF were officially reported from 2000 to 2019^*6*^. The Brazilian state with the highest number of cases was São Paulo, with 992 cases, followed by Santa Catarina with 457 cases^*6*^. While 476 cases in the São Paulo had a fatal outcome (48% fatality rate), no fatal case was reported in Santa Catarina^*6*^ ^,^ ^*7*^. These contrasting clinical outcomes corroborate the presence of two different spotted fever diseases-one reemerging with a high mortality rate and another emerging with a generally benign evolution.

Given the above factors and the historical narrative presented and in light of the clinical, epidemiological, and microbiological evidence, it is considered that there are two endemic spotted fever group rickettsioses under surveillance and subject to compulsory notification in Brazil-(i) BSF, caused by *R. rickettsii*, which manifests as a severe, potentially fatal disease and is transmitted mainly by the ticks *Amblyomma sculptum* and *A. aureolatum* ^2^ ^,^ ^3^ and (ii) an emerging spotted fever caused by *R. parkeri* sensu lato, which manifests as a benign acute disease and is transmitted primarily by *Amblyomma ovale* in parts of the Atlantic Forest biome of southern, southeastern, and northeastern regions and possibly by *A. tigrinum* in the Pampa biome of the southern region^4^ ^,^ ^5^ ^,^ ^8^ ^-^ ^11^.

Based on the above statements, the strategies that have been employed by the Brazilian Ministry of Health for the surveillance and release of epidemiological data regarding BSF need to be urgently revised since it currently considers all spotted fever cases and deaths, regardless of geographical origin in the country, as a single aggregation-BSF. Furthermore, the instruments (including the epidemiological investigation form and the configuration of the notification system) and the strategies used for investigation (the criteria for defining suspected and confirmed cases) in addition to the laboratory investigation protocols fundamentally apply only to BSF. An example is the inclusion of hemorrhagic manifestations (characteristic of *R. rickettsii* infection) as a constant clinical marker in the criteria for defining a suspected case, bearing in mind that such clinical presentation is not observed in cases of spotted fever caused by *R. parkeri* ^2^ ^-^ ^4^. In contrast, the occurrence of an inoculation eschar (a frequent clinical finding in *R. parkeri* infection) has been completely omitted from the definition criteria for surveillance purposes and the epidemiological investigation form.

The definition criteria and epidemiological information of another important rickettsiosis, murine typhus, which has been known to occur in Brazil since the last century, is also not included in the Brazilian rickettsial surveillance system^12^. Murine typhus is caused by *Rickettsia typhi,* transmitted by fleas, and a nationally notifiable disease. Maintenance of the same criteria for the notification, investigation, and final classification of different rickettsial diseases imposes several inaccuracies at both local and national levels, as with elementary epidemiological indicators such as incidence and fatality. In addition, the perception of the real distribution of each rickettsiosis becomes distorted throughout the country’s federal units.

In conclusion, it is necessary to urgently reassess the strategies and tools for the surveillance of rickettsioses in Brazil to consider the specificities of distinct diseases transmitted by specific vectors and, above all, caused by different species of *Rickettsia*. 

## References

[1] Fang R, Blanton LS, Walker DH (2017). Rickettsiae as emerging infectious agents. Clin Lab Med.

[2] de Oliveira SV, Guimarães JN, Reckziegel GC, Neves BM, Araújo-Vilges KM, Fonseca LX (2016). An update on the epidemiological situation of spotted fever in Brazil. J Venom Anim Toxins Incl Trop Dis.

[3] Angerami RN, da Silva AM, Nascimento EM, Colombo S, Wada MY, dos Santos FC (2009). Brazilian spotted fever: two faces of a same disease? A comparative study of clinical aspects between an old and a new endemic area in Brazil. Clin Microbiol Infect.

[4] Faccini-Martínez ÁA, Oliveira SV, Cerutti C, Labruna MB (2018). Febre maculosa por Rickettsia parkeri no Brasil: condutas de vigilância epidemiológica, diagnóstico e tratamento. J Health Biol Sci.

[5] Krawczak FS, Muñoz-Leal S, Guztzazky AC, Oliveira SV, Santos FC, Angerami RN (2016). Rickettsia sp. strain Atlantic rainforest infection in a patient from a spotted fever-endemic area in Southern Brazil. Am J Trop Med Hyg.

[6] Brasil. Ministério da Saúde. Secretaria de Vigilância em Saúde (2019). Casos confirmados de febre maculosa. Brasil, Grandes Regiões e Unidades Federadas. 2000 a 2019.

[7] Brasil. Ministério da Saúde. Secretaria de Vigilância em Saúde (2019). Óbitos de febre maculosa. Brasil, Grandes Regiões e Unidades Federadas. 2000-2019.

[8] Spolidorio MG, Labruna MB, Mantovani E, Brandao PE, Richtzenhain LJ, Yoshinari NH (2010). Novel spotted fever group rickettsiosis, Brazil. Emerg Infect Dis.

[9] Silva N, Eremeeva ME, Rozental T, Ribeiro GS, Paddock CD, Ramos EA (2011). Eschar-associated spotted fever rickettsiosis, Bahia, Brazil. Emerg Infect Dis.

[10] da Paixão Sevá A, Martins TF, Muñoz-Leal S, Rodrigues AC, Pinter A, Luz HR (2019). A human case of spotted fever caused by Rickettsia parkeri strain Atlantic rainforest and its association to the tick Amblyomma ovale. Parasit Vectors.

[11] Weck B, Krawczak FS, Costa FB, Dall'Agnol B, Marcili A, Reck J (2020). Rickettsia parkeri in the Pampa biome of southern Brazil: Isolation, molecular characterization, and serological evidence of canine infection. Vet Parasitol Reg Stud Reports.

[12] Silva LJ, Papaiordanou PM (2004). Murine (endemic) typhus in Brazil: case report and review. Rev Inst Med Trop Sao Paulo.

